# Bis{2-[(*E*)-(5-*tert*-butyl-2-hy­droxy­phen­yl)diazen­yl]benzoato}dimethyl­tin(IV)

**DOI:** 10.1107/S1600536811036543

**Published:** 2011-09-14

**Authors:** Tushar S. Basu Baul, Anup Paul, Edward R. T. Tiekink

**Affiliations:** a Department of Chemistry, North-Eastern Hill University, NEHU Permanent Campus, Umshing, Shillong 793 022, India; b Department of Chemistry, University of Malaya, 50603 Kuala Lumpur, Malaysia

## Abstract

In the title diorganotin dicarboxyl­ate, [Sn(CH_3_)_2_(C_17_H_17_N_2_O_3_)_2_], the tin(IV) atom is six-coordinated by four O atoms derived from asymmetrically coordinating carboxyl­ate ligands, and two methyl-C atoms. The resulting C_2_O_4_ donor set defines a skew-trapezoidal bipyramid with the Sn—C bonds disposed over the weaker Sn—O bonds. Within each carboxyl­ate ligand, the hydroxyl-H atom forms bifurcated O—H⋯(O,N) hydrogen bonds with carboxyl­ate-O and azo-N atoms. The dihedral angles between the benzene rings in the two ligands are 10.44 (11) and 34.24 (11)°. In the crystal, centrosymmetric dimers are formed through pairs of Sn⋯O inter­actions [2.8802 (16) Å], and the dimers are linked into supra­molecular layers in the *ac* plane by C—H⋯π inter­actions.

## Related literature

For background to the potential anti-cancer activity of related compounds, see: Basu Baul *et al.* (2011 ▶). For the synthesis of the ligand, see: Basu Baul *et al.* (2008 ▶). For related structural studies, see: Basu Baul *et al.* (2010 ▶). For a review of the structural chemistry of organotin carboxyl­ates, see: Tiekink (1991 ▶).
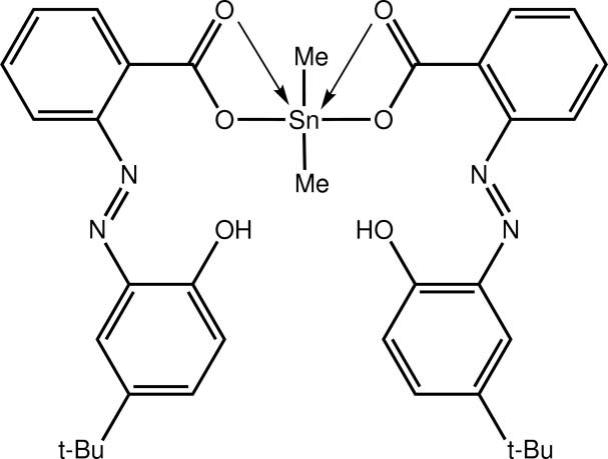


## Experimental

### 

#### Crystal data

[Sn(CH_3_)_2_(C_17_H_17_N_2_O_3_)_2_]
*M* *_r_* = 743.43
Monoclinic, 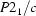

*a* = 9.6298 (1) Å
*b* = 31.8788 (4) Å
*c* = 11.0963 (1) Å
β = 93.502 (1)°
*V* = 3400.05 (6) Å^3^
*Z* = 4
Mo *K*α radiation
μ = 0.80 mm^−1^
*T* = 100 K
0.36 × 0.13 × 0.03 mm

#### Data collection

Bruker SMART APEXII diffractometer
Absorption correction: multi-scan (*SADABS*; Sheldrick, 1996 ▶) *T*_min_ = 0.895, *T*_max_ = 1
26762 measured reflections
7749 independent reflections
6165 reflections with *I* > 2σ(*I*)
*R*_int_ = 0.031

#### Refinement

*R*[*F* ^2^ > 2σ(*F* ^2^)] = 0.033
*wR*(*F* ^2^) = 0.071
*S* = 1.04
7749 reflections
434 parameters
H-atom parameters constrained
Δρ_max_ = 0.55 e Å^−3^
Δρ_min_ = −0.41 e Å^−3^

### 

Data collection: *APEX2* (Bruker, 2007 ▶); cell refinement: *SAINT* (Bruker, 2007 ▶); data reduction: *SAINT*; program(s) used to solve structure: *SHELXS86* (Sheldrick, 2008 ▶); program(s) used to refine structure: *SHELXL97* (Sheldrick, 2008 ▶); molecular graphics: *ORTEP-3* (Farrugia, 1997 ▶) and *DIAMOND* (Brandenburg, 2006 ▶); software used to prepare material for publication: *SHELXL97*.

## Supplementary Material

Crystal structure: contains datablock(s) general, I. DOI: 10.1107/S1600536811036543/hb6399sup1.cif

Structure factors: contains datablock(s) I. DOI: 10.1107/S1600536811036543/hb6399Isup2.hkl

Additional supplementary materials:  crystallographic information; 3D view; checkCIF report

## Figures and Tables

**Table 1:** Selected bond lengths (Å)

Sn—O1	2.1118 (16)
Sn—O2	2.6967 (16)
Sn—O4	2.1120 (16)
Sn—O5	2.4482 (16)
Sn—C35	2.081 (3)
Sn—C36	2.098 (2)

**Table 2:** Hydrogen-bond geometry (Å, °) *Cg*1 is the centroid of the C25–C30 ring.

*D*—H⋯*A*	*D*—H	H⋯*A*	*D*⋯*A*	*D*—H⋯*A*
O3—H3⋯O1	0.84	2.49	3.142 (2)	136
O3—H3⋯N1	0.84	1.87	2.573 (2)	140
O6—H6⋯O5	0.84	2.20	2.877 (3)	137
O6—H6⋯N3	0.84	1.93	2.620 (3)	139
C10—H10⋯*Cg*1^i^	0.95	2.97	3.863 (2)	157

Symmetry code: (i) 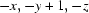.

**Fig. 1.**
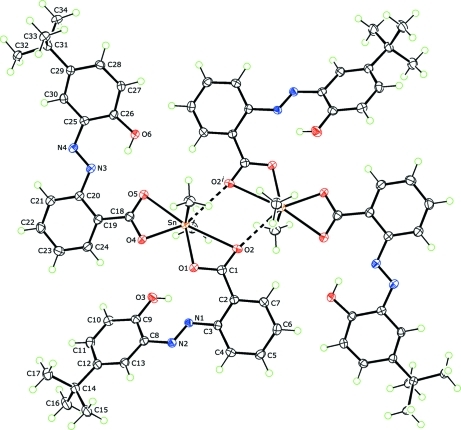
Molecular structure of (I) showing displacement ellipsoids at the 50% probability level. Centrosymmetrically related molecules associate via Sn···O interactions shown as dashed lines. Symmetry operation i: 1 - x, 1 - y, 1 - z.

**Fig. 2.**
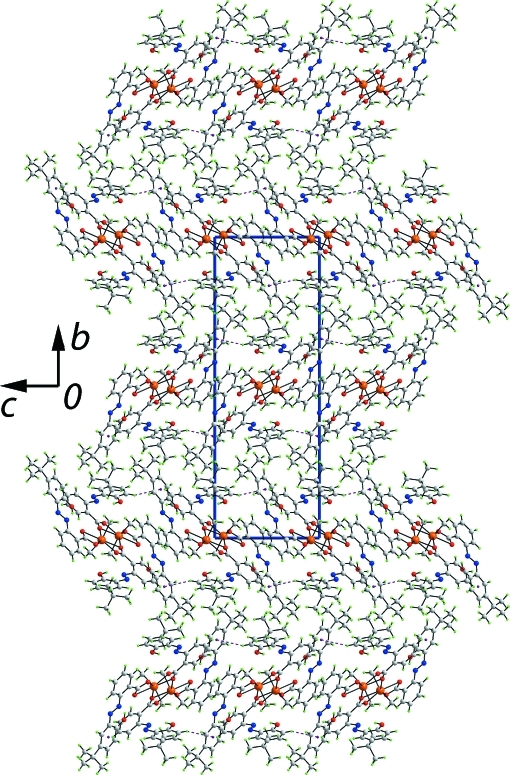
View in projection down the a axis of the crystal packing in (I), highlighting the stacking of supramolecular arrays sustained by C—H···π interactions shown as purple dashed lines.

## supplementary crystallographic information

### Comment

Organotin carboxylates related to the title compound, (I), have been investigated for potential anti-cancer activity (Basu Baul *et al.*, 2011). Complementing biological studies are structural investigations (Basu Baul *et al.*, 2010). In (I), the Sn atom is bound by two asymmetrically coordinating carboxylate ligands and two methyl groups, Fig. 1 and Table 1. The coordination geometry is based on a skew-trapezoidal bipyramid with the methyl groups disposed to lie over the weaker Sn—O bonds; the C35—Sn—C36 angle is 149.63 (10) °. The overall molecular conformation matches those normally observed for structures of the general formula *R*_2_Sn(O_2_CR')_2_ (Tiekink, 1991).

Centrosymmetrically related molecules associate into dimeric aggregates *via* weak Sn···O2^i^ contacts of 2.8802 (16) Å, Fig. 1, symmetry operation *i*: -*x*, 1 - *y*, -*z*. A consequence of this association is the significant lengthening of the Sn—O2 bond with respect to the chemically equivalent Sn—O5 bond, Table 1. The relative dispositions of the carboxylate residues are different in order to reduce steric hindrance. Thus, while the hydroxy group of the O1-carboxylate ligand is orientated towards the more strongly coordinating O1 atom, the hydroxy group of the O4-carboxylate ligand is orientated towards the weakly coordinating O5 atom, Fig. 1. Within each carboxylate ligand, intramolecular O—H···O,*N* hydrogen bonds are noted, Table 2. Despite these, the ligands exhibit significant deviations from planarity. The values of the O1—C1—C2—C3 and O4—C18—C19—C20 torsion angles of 15.2 (3) and 158.7 (2) °, respectively, indicate that the carboxylate groups lie out of the plane of the respective benzene ring to which it is attached. Significant twisting is found in the O1-carboxylate ligand with the dihedral angle formed between the two benzene rings being 34.24 (11) °. This arises in part to avoid a steric clash with a benzene ring of the adjacent carboxylate ligand. The O4-carboxylate ligand, being directed away from the rest of the molecule, is less twisted with the dihedral angle formed between the two benzene rings being 10.44 (11) °.

Over and above the intermolecular Sn···O interactions mentioned above, the most prominent feature of the crystal packing is the formation of C—H···π interactions, Table 2. These serve to link dimeric aggregates into supramolecular arrays in the *ac* plane. A view of the unit-cell contents is shown in Fig. 2 which highlights the stacking of layers along the *b* axis.

### Experimental

The title compound was prepared by reacting 2-[(*E*)-(5-*tert*-butyl-2-hydroxyphenyl)diazenyl]benzoic acid (Basu Baul *et al.*, 2008) (0.30 g, 1.00 mmol) and Me_2_SnO (0.08 g, 0.49 mmol) in anhydrous toluene (50 ml) using a Dean and Stark apparatus for 6 h. The red solution was filtered while hot, concentrated to one tenth of its initial volume and precipitated with hexane. The red precipitate was separated by filtration, washed with hexane (2 *x* 5 ml) and dried *in vacuo*. The dried residue was dissolved in chloroform-hexane (10:1 *v*/*v*) and filtered. The filtrate was allowed to evaporate at room temperature, which afforded red prisms. Yield: 0.15 g, 40%, *M*.pt. 439–441 K. Elemental analysis, found: C 58.44, H 5.61, N 7.37%. C_36_H_40_N_4_O_6_Sn requires: C 58.14, H, 5.43, N 7.54%. IR (KBr, cm^-1^): 1589 ν(OCO)*_asym_*. ^1^H-NMR (CDCl_3_, 400.44 MHz): δ H: 12.8 [br, 1H, OH], 8.22 [d, 8 Hz, 1H, H7], 7.92 [d, 8 Hz, 1H, H4], 7.78 [d, 2.5 Hz, 1H, H13], 7.60 [t, 8 Hz, 1H, H5], 7.50 [t, 8 Hz, 1H, H6], 7.37 [dd, 2.5, 8 Hz, 1H, H11], 6.96 [d, 8 Hz, 1H, H10], 1.32 [s, 9H, CH_3_], 1.19 [s, 3H, Sn—CH_3_] p.p.m. ^119^Sn-NMR (CDCl_3_, 149.33): δ -112.7 p.p.m.

### Refinement

All H-atoms were placed in calculated positions (O—H = 0.84 Å, and C—H = 0.95–0.98 Å) and were included in the refinement in the riding model approximation with *U*_iso_(H) set to 1.2–1.5*U*_eq_(carrier atom).

### Crystal data

**Table:**

[Sn(CH_3_)_2_(C_17_H_17_N_2_O_3_)_2_]	*F*(000) = 1528
*M**_r_* = 743.43	*D*_x_ = 1.452 Mg m^−^^3^
Monoclinic, *P*2_1_/*c*	Mo *K*α radiation, λ = 0.71073 Å
Hall symbol: -P 2ybc	Cell parameters from 9596 reflections
*a* = 9.6298 (1) Å	θ = 2.5–27.5°
*b* = 31.8788 (4) Å	µ = 0.80 mm^−^^1^
*c* = 11.0963 (1) Å	*T* = 100 K
β = 93.502 (1)°	Prism, red
*V* = 3400.05 (6) Å^3^	0.36 × 0.13 × 0.03 mm
*Z* = 4	

### Data collection

**Table:**

Bruker SMART APEXII diffractometer	7749 independent reflections
Radiation source: sealed tube	6165 reflections with *I* > 2σ(*I*)
graphite	*R*_int_ = 0.031
φ and ω scans	θ_max_ = 27.5°, θ_min_ = 2.0°
Absorption correction: multi-scan (*SADABS*; Sheldrick, 1996)	*h* = −12→12
*T*_min_ = 0.895, *T*_max_ = 1	*k* = −41→31
26762 measured reflections	*l* = −13→14

### Refinement

**Table:**

Refinement on *F*^2^	Primary atom site location: structure-invariant direct methods
Least-squares matrix: full	Secondary atom site location: difference Fourier map
*R*[*F*^2^ > 2σ(*F*^2^)] = 0.033	Hydrogen site location: inferred from neighbouring sites
*wR*(*F*^2^) = 0.071	H-atom parameters constrained
*S* = 1.04	*w* = 1/[σ^2^(*F*_o_^2^) + (0.0302*P*)^2^ + 2.0032*P*] where *P* = (*F*_o_^2^ + 2*F*_c_^2^)/3
7749 reflections	(Δ/σ)_max_ = 0.001
434 parameters	Δρ_max_ = 0.55 e Å^−^^3^
0 restraints	Δρ_min_ = −0.41 e Å^−^^3^

### Special details

**Table:**

Geometry. All s.u.'s (except the s.u. in the dihedral angle between two l.s. planes) are estimated using the full covariance matrix. The cell s.u.'s are taken into account individually in the estimation of s.u.'s in distances, angles and torsion angles; correlations between s.u.'s in cell parameters are only used when they are defined by crystal symmetry. An approximate (isotropic) treatment of cell s.u.'s is used for estimating s.u.'s involving l.s. planes.
Refinement. Refinement of *F*^2^ against ALL reflections. The weighted *R*-factor *wR* and goodness of fit *S* are based on *F*^2^, conventional *R*-factors *R* are based on *F*, with *F* set to zero for negative *F*^2^. The threshold expression of *F*^2^ > 2σ(*F*^2^) is used only for calculating *R*-factors(gt) *etc*. and is not relevant to the choice of reflections for refinement. *R*-factors based on *F*^2^ are statistically about twice as large as those based on *F*, and *R*- factors based on ALL data will be even larger.

### Fractional atomic coordinates and isotropic or equivalent isotropic displacement parameters (Å^2^)

**Table:**

	*x*	*y*	*z*	*U*_iso_*/*U*_eq_	
Sn	0.277358 (16)	0.493245 (5)	0.417857 (14)	0.01732 (5)	
O1	0.23555 (16)	0.45124 (6)	0.55829 (14)	0.0217 (4)	
O2	0.45495 (17)	0.46778 (6)	0.60060 (14)	0.0216 (4)	
O3	0.08900 (17)	0.37965 (6)	0.40295 (14)	0.0236 (4)	
H3	0.1412	0.3856	0.4639	0.035*	
O4	0.06960 (16)	0.47867 (6)	0.36115 (14)	0.0214 (4)	
O5	0.16032 (17)	0.52164 (6)	0.23347 (14)	0.0217 (4)	
O6	0.18551 (16)	0.60409 (6)	0.13158 (15)	0.0216 (4)	
H6	0.1378	0.5823	0.1394	0.032*	
N1	0.15350 (19)	0.37784 (6)	0.63135 (17)	0.0165 (4)	
N2	0.03907 (19)	0.36266 (6)	0.65957 (17)	0.0175 (4)	
N3	−0.0403 (2)	0.55980 (6)	0.08342 (16)	0.0180 (4)	
N4	−0.0860 (2)	0.58421 (7)	0.00045 (16)	0.0177 (4)	
C1	0.3515 (2)	0.44669 (8)	0.6217 (2)	0.0180 (5)	
C2	0.3565 (2)	0.41453 (8)	0.71981 (19)	0.0163 (5)	
C3	0.2554 (2)	0.38306 (8)	0.72847 (19)	0.0157 (5)	
C4	0.2650 (2)	0.35474 (8)	0.8236 (2)	0.0187 (5)	
H4	0.1963	0.3336	0.8294	0.022*	
C5	0.3747 (2)	0.35728 (8)	0.9100 (2)	0.0213 (5)	
H5	0.3800	0.3382	0.9758	0.026*	
C6	0.4769 (2)	0.38759 (8)	0.9010 (2)	0.0219 (5)	
H6A	0.5530	0.3889	0.9596	0.026*	
C7	0.4674 (2)	0.41596 (8)	0.8063 (2)	0.0192 (5)	
H7	0.5375	0.4367	0.8003	0.023*	
C8	−0.0562 (2)	0.35492 (8)	0.5600 (2)	0.0164 (5)	
C9	−0.0301 (2)	0.36295 (8)	0.4384 (2)	0.0176 (5)	
C10	−0.1336 (2)	0.35283 (8)	0.3498 (2)	0.0200 (5)	
H10	−0.1185	0.3579	0.2673	0.024*	
C11	−0.2576 (2)	0.33549 (8)	0.3808 (2)	0.0208 (5)	
H11	−0.3260	0.3287	0.3186	0.025*	
C12	−0.2865 (2)	0.32747 (8)	0.5013 (2)	0.0180 (5)	
C13	−0.1839 (2)	0.33798 (8)	0.5883 (2)	0.0185 (5)	
H13	−0.2009	0.3335	0.6707	0.022*	
C14	−0.4276 (2)	0.30919 (8)	0.5294 (2)	0.0210 (5)	
C15	−0.4387 (3)	0.30246 (10)	0.6645 (2)	0.0301 (6)	
H15A	−0.5312	0.2915	0.6793	0.045*	
H15B	−0.4242	0.3292	0.7069	0.045*	
H15C	−0.3677	0.2823	0.6942	0.045*	
C16	−0.4500 (3)	0.26688 (9)	0.4652 (3)	0.0303 (6)	
H16A	−0.3785	0.2470	0.4956	0.046*	
H16B	−0.4435	0.2707	0.3781	0.046*	
H16C	−0.5422	0.2559	0.4810	0.046*	
C17	−0.5426 (3)	0.33979 (9)	0.4843 (2)	0.0246 (6)	
H17A	−0.6334	0.3287	0.5038	0.037*	
H17B	−0.5403	0.3432	0.3967	0.037*	
H17C	−0.5275	0.3671	0.5237	0.037*	
C18	0.0605 (2)	0.49959 (8)	0.2613 (2)	0.0190 (5)	
C19	−0.0703 (2)	0.49363 (8)	0.1842 (2)	0.0182 (5)	
C20	−0.1186 (2)	0.52255 (8)	0.09590 (19)	0.0163 (5)	
C21	−0.2430 (2)	0.51461 (8)	0.0285 (2)	0.0203 (5)	
H21	−0.2772	0.5343	−0.0303	0.024*	
C22	−0.3161 (3)	0.47835 (9)	0.0468 (2)	0.0236 (6)	
H22	−0.4002	0.4730	−0.0001	0.028*	
C23	−0.2682 (3)	0.44941 (9)	0.1332 (2)	0.0242 (6)	
H23	−0.3191	0.4244	0.1454	0.029*	
C24	−0.1463 (2)	0.45724 (8)	0.2012 (2)	0.0216 (5)	
H24	−0.1137	0.4375	0.2605	0.026*	
C25	−0.0119 (2)	0.62188 (8)	−0.00658 (19)	0.0164 (5)	
C26	0.1145 (2)	0.63123 (8)	0.0583 (2)	0.0174 (5)	
C27	0.1705 (2)	0.67117 (8)	0.0446 (2)	0.0205 (5)	
H27	0.2574	0.6780	0.0851	0.025*	
C28	0.1019 (2)	0.70090 (8)	−0.0266 (2)	0.0191 (5)	
H28	0.1415	0.7281	−0.0316	0.023*	
C29	−0.0242 (2)	0.69246 (8)	−0.09212 (19)	0.0155 (5)	
C30	−0.0765 (2)	0.65243 (8)	−0.08173 (19)	0.0167 (5)	
H30	−0.1598	0.6453	−0.1274	0.020*	
C31	−0.0990 (2)	0.72721 (8)	−0.1662 (2)	0.0176 (5)	
C32	−0.2250 (2)	0.71042 (8)	−0.2421 (2)	0.0235 (5)	
H32A	−0.2927	0.6988	−0.1887	0.035*	
H32B	−0.1949	0.6884	−0.2962	0.035*	
H32C	−0.2682	0.7333	−0.2900	0.035*	
C33	−0.1492 (3)	0.76023 (8)	−0.0787 (2)	0.0217 (5)	
H33A	−0.1977	0.7827	−0.1244	0.033*	
H33B	−0.0691	0.7719	−0.0313	0.033*	
H33C	−0.2130	0.7472	−0.0242	0.033*	
C34	0.0008 (2)	0.74802 (8)	−0.2511 (2)	0.0206 (5)	
H34A	−0.0475	0.7708	−0.2955	0.031*	
H34B	0.0322	0.7272	−0.3083	0.031*	
H34C	0.0814	0.7593	−0.2035	0.031*	
C35	0.2588 (3)	0.55181 (8)	0.4982 (2)	0.0254 (6)	
H35A	0.3164	0.5526	0.5741	0.038*	
H35B	0.1614	0.5568	0.5147	0.038*	
H35C	0.2900	0.5736	0.4437	0.038*	
C36	0.3840 (3)	0.45265 (8)	0.3069 (2)	0.0247 (6)	
H36A	0.4412	0.4691	0.2541	0.037*	
H36B	0.3167	0.4359	0.2575	0.037*	
H36C	0.4439	0.4339	0.3571	0.037*	

### Atomic displacement parameters (Å^2^)

**Table:**

	*U*^11^	*U*^22^	*U*^33^	*U*^12^	*U*^13^	*U*^23^
Sn	0.01744 (8)	0.01519 (9)	0.01906 (8)	−0.00012 (7)	−0.00111 (5)	0.00331 (7)
O1	0.0198 (9)	0.0215 (10)	0.0234 (9)	−0.0022 (7)	−0.0033 (7)	0.0076 (7)
O2	0.0215 (9)	0.0198 (10)	0.0234 (9)	−0.0055 (7)	0.0016 (7)	0.0046 (7)
O3	0.0213 (9)	0.0288 (11)	0.0209 (9)	−0.0067 (8)	0.0034 (7)	−0.0011 (8)
O4	0.0198 (8)	0.0237 (10)	0.0202 (8)	−0.0014 (7)	−0.0034 (6)	0.0076 (7)
O5	0.0186 (8)	0.0233 (10)	0.0230 (8)	−0.0011 (7)	−0.0016 (7)	0.0054 (7)
O6	0.0180 (8)	0.0192 (10)	0.0269 (9)	−0.0003 (7)	−0.0045 (7)	0.0059 (7)
N1	0.0160 (10)	0.0126 (11)	0.0207 (10)	−0.0010 (8)	0.0000 (7)	0.0002 (8)
N2	0.0162 (10)	0.0158 (12)	0.0205 (10)	0.0001 (8)	0.0010 (8)	0.0002 (8)
N3	0.0196 (10)	0.0169 (12)	0.0172 (10)	0.0029 (8)	−0.0004 (8)	0.0013 (8)
N4	0.0199 (10)	0.0180 (12)	0.0150 (9)	0.0027 (8)	0.0011 (7)	0.0008 (8)
C1	0.0200 (12)	0.0168 (14)	0.0171 (11)	−0.0002 (10)	0.0014 (9)	−0.0014 (9)
C2	0.0176 (11)	0.0144 (13)	0.0170 (11)	0.0010 (9)	0.0017 (9)	−0.0004 (9)
C3	0.0141 (11)	0.0169 (14)	0.0164 (11)	0.0021 (9)	0.0016 (8)	−0.0010 (9)
C4	0.0183 (12)	0.0179 (14)	0.0202 (11)	−0.0027 (10)	0.0041 (9)	0.0000 (10)
C5	0.0232 (12)	0.0237 (15)	0.0171 (11)	0.0019 (10)	0.0017 (9)	0.0040 (10)
C6	0.0190 (12)	0.0273 (16)	0.0188 (12)	0.0014 (10)	−0.0028 (9)	−0.0013 (10)
C7	0.0180 (12)	0.0196 (14)	0.0201 (11)	−0.0025 (10)	0.0011 (9)	−0.0010 (10)
C8	0.0170 (11)	0.0126 (13)	0.0197 (11)	0.0031 (9)	0.0010 (9)	−0.0005 (9)
C9	0.0183 (11)	0.0132 (13)	0.0215 (12)	0.0010 (9)	0.0030 (9)	−0.0009 (9)
C10	0.0251 (13)	0.0195 (14)	0.0155 (11)	0.0013 (10)	0.0030 (9)	−0.0032 (9)
C11	0.0204 (12)	0.0203 (15)	0.0212 (12)	0.0011 (10)	−0.0027 (9)	−0.0060 (10)
C12	0.0154 (11)	0.0145 (14)	0.0239 (12)	0.0022 (9)	0.0013 (9)	−0.0017 (10)
C13	0.0172 (11)	0.0188 (14)	0.0194 (11)	0.0029 (10)	0.0016 (9)	0.0000 (10)
C14	0.0161 (12)	0.0210 (15)	0.0257 (12)	−0.0005 (10)	0.0003 (9)	−0.0021 (10)
C15	0.0187 (12)	0.0437 (19)	0.0281 (14)	−0.0076 (12)	0.0015 (10)	0.0069 (12)
C16	0.0184 (13)	0.0252 (17)	0.0471 (16)	−0.0022 (11)	0.0003 (12)	−0.0053 (13)
C17	0.0198 (12)	0.0253 (16)	0.0288 (13)	0.0023 (11)	0.0007 (10)	−0.0038 (11)
C18	0.0195 (11)	0.0175 (15)	0.0200 (11)	0.0025 (10)	0.0008 (9)	0.0000 (9)
C19	0.0193 (11)	0.0197 (13)	0.0157 (10)	0.0018 (10)	0.0005 (8)	0.0001 (10)
C20	0.0185 (11)	0.0167 (13)	0.0138 (10)	0.0024 (9)	0.0023 (8)	−0.0011 (9)
C21	0.0231 (12)	0.0188 (15)	0.0188 (11)	0.0019 (10)	−0.0007 (9)	−0.0010 (10)
C22	0.0218 (12)	0.0262 (15)	0.0221 (12)	−0.0020 (11)	−0.0040 (9)	−0.0023 (11)
C23	0.0259 (13)	0.0224 (15)	0.0244 (12)	−0.0053 (11)	0.0027 (10)	0.0005 (11)
C24	0.0235 (12)	0.0215 (15)	0.0196 (12)	0.0016 (10)	−0.0004 (9)	0.0038 (10)
C25	0.0179 (11)	0.0161 (15)	0.0154 (10)	0.0014 (9)	0.0028 (8)	−0.0004 (9)
C26	0.0156 (11)	0.0191 (14)	0.0176 (11)	0.0043 (9)	0.0010 (9)	0.0014 (9)
C27	0.0150 (11)	0.0238 (15)	0.0224 (12)	−0.0012 (10)	−0.0022 (9)	0.0012 (10)
C28	0.0207 (12)	0.0160 (14)	0.0206 (12)	−0.0028 (10)	0.0008 (9)	0.0000 (10)
C29	0.0153 (11)	0.0192 (14)	0.0122 (10)	0.0013 (9)	0.0018 (8)	−0.0003 (9)
C30	0.0147 (11)	0.0220 (14)	0.0134 (10)	0.0000 (9)	0.0008 (8)	−0.0013 (9)
C31	0.0166 (11)	0.0181 (14)	0.0178 (11)	−0.0004 (9)	−0.0006 (9)	0.0017 (9)
C32	0.0244 (13)	0.0212 (15)	0.0239 (12)	0.0008 (10)	−0.0064 (10)	0.0041 (10)
C33	0.0235 (13)	0.0203 (15)	0.0213 (12)	0.0030 (10)	0.0017 (9)	0.0008 (10)
C34	0.0260 (12)	0.0148 (15)	0.0211 (11)	0.0024 (10)	0.0029 (9)	0.0026 (10)
C35	0.0271 (13)	0.0199 (15)	0.0292 (14)	0.0030 (11)	0.0027 (10)	−0.0013 (11)
C36	0.0256 (13)	0.0215 (15)	0.0266 (13)	0.0019 (11)	−0.0012 (10)	−0.0032 (11)

### Geometric parameters (Å, °)

**Table:**

Sn—O1	2.1118 (16)	C15—H15C	0.9800
Sn—O2	2.6967 (16)	C16—H16A	0.9800
Sn—O4	2.1120 (16)	C16—H16B	0.9800
Sn—O5	2.4482 (16)	C16—H16C	0.9800
Sn—C35	2.081 (3)	C17—H17A	0.9800
Sn—C36	2.098 (2)	C17—H17B	0.9800
Sn—O2^i^	2.8802 (16)	C17—H17C	0.9800
O1—C1	1.291 (3)	C18—C19	1.491 (3)
O2—C1	1.236 (3)	C19—C24	1.391 (3)
O3—C9	1.345 (3)	C19—C20	1.404 (3)
O3—H3	0.8400	C20—C21	1.396 (3)
O4—C18	1.291 (3)	C21—C22	1.375 (4)
O5—C18	1.245 (3)	C21—H21	0.9500
O6—C26	1.345 (3)	C22—C23	1.389 (4)
O6—H6	0.8400	C22—H22	0.9500
N1—N2	1.260 (3)	C23—C24	1.379 (3)
N1—C3	1.422 (3)	C23—H23	0.9500
N2—C8	1.414 (3)	C24—H24	0.9500
N3—N4	1.264 (3)	C25—C30	1.403 (3)
N3—C20	1.418 (3)	C25—C26	1.408 (3)
N4—C25	1.402 (3)	C26—C27	1.395 (3)
C1—C2	1.494 (3)	C27—C28	1.377 (3)
C2—C7	1.392 (3)	C27—H27	0.9500
C2—C3	1.405 (3)	C28—C29	1.403 (3)
C3—C4	1.388 (3)	C28—H28	0.9500
C4—C5	1.385 (3)	C29—C30	1.379 (3)
C4—H4	0.9500	C29—C31	1.533 (3)
C5—C6	1.387 (3)	C30—H30	0.9500
C5—H5	0.9500	C31—C33	1.530 (3)
C6—C7	1.385 (3)	C31—C32	1.531 (3)
C6—H6A	0.9500	C31—C34	1.536 (3)
C7—H7	0.9500	C32—H32A	0.9800
C8—C13	1.397 (3)	C32—H32B	0.9800
C8—C9	1.411 (3)	C32—H32C	0.9800
C9—C10	1.394 (3)	C33—H33A	0.9800
C10—C11	1.379 (3)	C33—H33B	0.9800
C10—H10	0.9500	C33—H33C	0.9800
C11—C12	1.405 (3)	C34—H34A	0.9800
C11—H11	0.9500	C34—H34B	0.9800
C12—C13	1.379 (3)	C34—H34C	0.9800
C12—C14	1.529 (3)	C35—H35A	0.9800
C13—H13	0.9500	C35—H35B	0.9800
C14—C15	1.525 (3)	C35—H35C	0.9800
C14—C16	1.534 (4)	C36—H36A	0.9800
C14—C17	1.536 (3)	C36—H36B	0.9800
C15—H15A	0.9800	C36—H36C	0.9800
C15—H15B	0.9800		
			
C35—Sn—C36	149.63 (10)	C14—C16—H16B	109.5
C35—Sn—O4	102.74 (9)	H16A—C16—H16B	109.5
C36—Sn—O4	100.26 (8)	C14—C16—H16C	109.5
C35—Sn—O1	103.14 (9)	H16A—C16—H16C	109.5
C36—Sn—O1	99.46 (9)	H16B—C16—H16C	109.5
O4—Sn—O1	81.98 (6)	C14—C17—H17A	109.5
C35—Sn—O5	88.82 (8)	C14—C17—H17B	109.5
C36—Sn—O5	87.38 (8)	H17A—C17—H17B	109.5
O4—Sn—O5	56.84 (6)	C14—C17—H17C	109.5
O1—Sn—O5	138.79 (6)	H17A—C17—H17C	109.5
C35—Sn—C18	97.36 (9)	H17B—C17—H17C	109.5
C36—Sn—C18	93.25 (8)	O5—C18—O4	119.5 (2)
O4—Sn—C18	28.89 (7)	O5—C18—C19	124.6 (2)
O1—Sn—C18	110.80 (7)	O4—C18—C19	115.9 (2)
O5—Sn—C18	27.98 (6)	O5—C18—Sn	67.37 (12)
C35—Sn—O2	90.85 (8)	O4—C18—Sn	52.20 (11)
C36—Sn—O2	86.85 (8)	C19—C18—Sn	166.89 (17)
O4—Sn—O2	134.70 (6)	C24—C19—C20	119.2 (2)
O1—Sn—O2	52.77 (5)	C24—C19—C18	117.5 (2)
O5—Sn—O2	168.00 (5)	C20—C19—C18	123.3 (2)
C18—Sn—O2	163.18 (6)	C21—C20—C19	119.5 (2)
C35—Sn—O2^i^	75.17 (8)	C21—C20—N3	122.9 (2)
C36—Sn—O2^i^	75.64 (8)	C19—C20—N3	117.6 (2)
O4—Sn—O2^i^	155.42 (5)	C22—C21—C20	120.3 (2)
O1—Sn—O2^i^	122.54 (5)	C22—C21—H21	119.9
O5—Sn—O2^i^	98.59 (5)	C20—C21—H21	119.9
C18—Sn—O2^i^	126.53 (6)	C21—C22—C23	120.5 (2)
O2—Sn—O2^i^	69.77 (5)	C21—C22—H22	119.7
C1—O1—Sn	106.00 (14)	C23—C22—H22	119.7
C1—O2—Sn	79.89 (13)	C24—C23—C22	119.6 (2)
C9—O3—H3	109.5	C24—C23—H23	120.2
C18—O4—Sn	98.92 (14)	C22—C23—H23	120.2
C18—O5—Sn	84.64 (13)	C23—C24—C19	120.9 (2)
C26—O6—H6	109.5	C23—C24—H24	119.5
N2—N1—C3	115.46 (18)	C19—C24—H24	119.5
N1—N2—C8	114.07 (18)	N4—C25—C30	114.8 (2)
N4—N3—C20	115.05 (19)	N4—C25—C26	125.5 (2)
N3—N4—C25	114.36 (19)	C30—C25—C26	119.7 (2)
O2—C1—O1	121.1 (2)	O6—C26—C27	117.8 (2)
O2—C1—C2	121.4 (2)	O6—C26—C25	124.4 (2)
O1—C1—C2	117.5 (2)	C27—C26—C25	117.8 (2)
C7—C2—C3	118.8 (2)	C28—C27—C26	121.0 (2)
C7—C2—C1	118.0 (2)	C28—C27—H27	119.5
C3—C2—C1	123.3 (2)	C26—C27—H27	119.5
C4—C3—C2	120.2 (2)	C27—C28—C29	122.4 (2)
C4—C3—N1	120.7 (2)	C27—C28—H28	118.8
C2—C3—N1	118.7 (2)	C29—C28—H28	118.8
C5—C4—C3	120.1 (2)	C30—C29—C28	116.3 (2)
C5—C4—H4	120.0	C30—C29—C31	123.4 (2)
C3—C4—H4	120.0	C28—C29—C31	120.2 (2)
C4—C5—C6	120.4 (2)	C29—C30—C25	122.7 (2)
C4—C5—H5	119.8	C29—C30—H30	118.6
C6—C5—H5	119.8	C25—C30—H30	118.6
C7—C6—C5	119.7 (2)	C33—C31—C32	108.61 (19)
C7—C6—H6A	120.2	C33—C31—C29	108.23 (18)
C5—C6—H6A	120.2	C32—C31—C29	111.9 (2)
C6—C7—C2	120.9 (2)	C33—C31—C34	109.0 (2)
C6—C7—H7	119.5	C32—C31—C34	108.57 (19)
C2—C7—H7	119.5	C29—C31—C34	110.45 (18)
C13—C8—C9	119.7 (2)	C31—C32—H32A	109.5
C13—C8—N2	115.5 (2)	C31—C32—H32B	109.5
C9—C8—N2	124.8 (2)	H32A—C32—H32B	109.5
O3—C9—C10	118.1 (2)	C31—C32—H32C	109.5
O3—C9—C8	123.8 (2)	H32A—C32—H32C	109.5
C10—C9—C8	118.1 (2)	H32B—C32—H32C	109.5
C11—C10—C9	120.6 (2)	C31—C33—H33A	109.5
C11—C10—H10	119.7	C31—C33—H33B	109.5
C9—C10—H10	119.7	H33A—C33—H33B	109.5
C10—C11—C12	122.4 (2)	C31—C33—H33C	109.5
C10—C11—H11	118.8	H33A—C33—H33C	109.5
C12—C11—H11	118.8	H33B—C33—H33C	109.5
C13—C12—C11	116.5 (2)	C31—C34—H34A	109.5
C13—C12—C14	123.9 (2)	C31—C34—H34B	109.5
C11—C12—C14	119.6 (2)	H34A—C34—H34B	109.5
C12—C13—C8	122.6 (2)	C31—C34—H34C	109.5
C12—C13—H13	118.7	H34A—C34—H34C	109.5
C8—C13—H13	118.7	H34B—C34—H34C	109.5
C15—C14—C12	111.73 (19)	Sn—C35—H35A	109.5
C15—C14—C16	108.4 (2)	Sn—C35—H35B	109.5
C12—C14—C16	110.0 (2)	H35A—C35—H35B	109.5
C15—C14—C17	108.5 (2)	Sn—C35—H35C	109.5
C12—C14—C17	108.9 (2)	H35A—C35—H35C	109.5
C16—C14—C17	109.2 (2)	H35B—C35—H35C	109.5
C14—C15—H15A	109.5	Sn—C36—H36A	109.5
C14—C15—H15B	109.5	Sn—C36—H36B	109.5
H15A—C15—H15B	109.5	H36A—C36—H36B	109.5
C14—C15—H15C	109.5	Sn—C36—H36C	109.5
H15A—C15—H15C	109.5	H36A—C36—H36C	109.5
H15B—C15—H15C	109.5	H36B—C36—H36C	109.5
C14—C16—H16A	109.5		
			
C35—Sn—O1—C1	−83.78 (16)	C11—C12—C14—C16	59.1 (3)
C36—Sn—O1—C1	75.78 (16)	C13—C12—C14—C17	117.6 (3)
O4—Sn—O1—C1	174.93 (16)	C11—C12—C14—C17	−60.6 (3)
O5—Sn—O1—C1	172.67 (13)	Sn—O5—C18—O4	3.3 (2)
C18—Sn—O1—C1	172.96 (15)	Sn—O5—C18—C19	−173.8 (2)
O2—Sn—O1—C1	−2.88 (13)	Sn—O4—C18—O5	−3.9 (2)
O2^i^—Sn—O1—C1	−3.15 (17)	Sn—O4—C18—C19	173.52 (17)
C35—Sn—O2—C1	108.86 (15)	C35—Sn—C18—O5	73.29 (15)
C36—Sn—O2—C1	−101.45 (16)	C36—Sn—C18—O5	−78.19 (15)
O4—Sn—O2—C1	−0.12 (17)	O4—Sn—C18—O5	176.4 (2)
O1—Sn—O2—C1	2.94 (14)	O1—Sn—C18—O5	−179.59 (13)
O5—Sn—O2—C1	−162.8 (3)	O2—Sn—C18—O5	−168.08 (17)
C18—Sn—O2—C1	−10.6 (3)	O2^i^—Sn—C18—O5	−3.68 (17)
O2^i^—Sn—O2—C1	−177.31 (17)	C35—Sn—C18—O4	−103.08 (15)
C35—Sn—O4—C18	82.07 (16)	C36—Sn—C18—O4	105.44 (15)
C36—Sn—O4—C18	−77.96 (16)	O1—Sn—C18—O4	4.04 (16)
O1—Sn—O4—C18	−176.19 (15)	O5—Sn—C18—O4	−176.4 (2)
O5—Sn—O4—C18	2.03 (13)	O2—Sn—C18—O4	15.6 (3)
O2—Sn—O4—C18	−173.73 (12)	O2^i^—Sn—C18—O4	179.96 (12)
O2^i^—Sn—O4—C18	−0.1 (2)	C35—Sn—C18—C19	−129.7 (7)
C35—Sn—O5—C18	−108.18 (15)	C36—Sn—C18—C19	78.9 (7)
C36—Sn—O5—C18	101.96 (16)	O4—Sn—C18—C19	−26.6 (7)
O4—Sn—O5—C18	−2.09 (13)	O1—Sn—C18—C19	−22.6 (7)
O1—Sn—O5—C18	0.57 (18)	O5—Sn—C18—C19	157.0 (8)
O2—Sn—O5—C18	163.3 (2)	O2—Sn—C18—C19	−11.0 (9)
O2^i^—Sn—O5—C18	177.01 (14)	O2^i^—Sn—C18—C19	153.4 (7)
C3—N1—N2—C8	−175.48 (19)	O5—C18—C19—C24	155.3 (2)
C20—N3—N4—C25	176.77 (18)	O4—C18—C19—C24	−21.9 (3)
Sn—O2—C1—O1	−4.5 (2)	Sn—C18—C19—C24	1.2 (8)
Sn—O2—C1—C2	174.5 (2)	O5—C18—C19—C20	−24.1 (4)
Sn—O1—C1—O2	5.9 (3)	O4—C18—C19—C20	158.7 (2)
Sn—O1—C1—C2	−173.12 (16)	Sn—C18—C19—C20	−178.2 (6)
O2—C1—C2—C7	16.0 (3)	C24—C19—C20—C21	1.2 (3)
O1—C1—C2—C7	−165.0 (2)	C18—C19—C20—C21	−179.5 (2)
O2—C1—C2—C3	−163.7 (2)	C24—C19—C20—N3	178.4 (2)
O1—C1—C2—C3	15.2 (3)	C18—C19—C20—N3	−2.3 (3)
C7—C2—C3—C4	1.6 (3)	N4—N3—C20—C21	−4.8 (3)
C1—C2—C3—C4	−178.6 (2)	N4—N3—C20—C19	178.1 (2)
C7—C2—C3—N1	−171.1 (2)	C19—C20—C21—C22	−1.3 (3)
C1—C2—C3—N1	8.6 (3)	N3—C20—C21—C22	−178.3 (2)
N2—N1—C3—C4	34.5 (3)	C20—C21—C22—C23	0.7 (4)
N2—N1—C3—C2	−152.8 (2)	C21—C22—C23—C24	0.1 (4)
C2—C3—C4—C5	−0.3 (3)	C22—C23—C24—C19	−0.2 (4)
N1—C3—C4—C5	172.3 (2)	C20—C19—C24—C23	−0.4 (4)
C3—C4—C5—C6	−1.1 (4)	C18—C19—C24—C23	−179.8 (2)
C4—C5—C6—C7	1.3 (4)	N3—N4—C25—C30	−168.2 (2)
C5—C6—C7—C2	0.1 (4)	N3—N4—C25—C26	8.9 (3)
C3—C2—C7—C6	−1.5 (3)	N4—C25—C26—O6	3.8 (4)
C1—C2—C7—C6	178.7 (2)	C30—C25—C26—O6	−179.2 (2)
N1—N2—C8—C13	179.8 (2)	N4—C25—C26—C27	−176.9 (2)
N1—N2—C8—C9	0.0 (3)	C30—C25—C26—C27	0.0 (3)
C13—C8—C9—O3	179.2 (2)	O6—C26—C27—C28	−178.4 (2)
N2—C8—C9—O3	−1.0 (4)	C25—C26—C27—C28	2.3 (3)
C13—C8—C9—C10	−1.1 (4)	C26—C27—C28—C29	−2.3 (4)
N2—C8—C9—C10	178.7 (2)	C27—C28—C29—C30	−0.2 (3)
O3—C9—C10—C11	179.8 (2)	C27—C28—C29—C31	177.6 (2)
C8—C9—C10—C11	0.0 (4)	C28—C29—C30—C25	2.5 (3)
C9—C10—C11—C12	0.5 (4)	C31—C29—C30—C25	−175.2 (2)
C10—C11—C12—C13	0.2 (4)	N4—C25—C30—C29	174.8 (2)
C10—C11—C12—C14	178.5 (2)	C26—C25—C30—C29	−2.5 (3)
C11—C12—C13—C8	−1.3 (4)	C30—C29—C31—C33	110.7 (2)
C14—C12—C13—C8	−179.5 (2)	C28—C29—C31—C33	−67.0 (3)
C9—C8—C13—C12	1.8 (4)	C30—C29—C31—C32	−9.0 (3)
N2—C8—C13—C12	−178.0 (2)	C28—C29—C31—C32	173.4 (2)
C13—C12—C14—C15	−2.2 (3)	C30—C29—C31—C34	−130.1 (2)
C11—C12—C14—C15	179.6 (2)	C28—C29—C31—C34	52.3 (3)
C13—C12—C14—C16	−122.7 (3)		

Symmetry codes: (i) −*x*+1, −*y*+1, −*z*+1.

### Hydrogen-bond geometry (Å, °)

**Table:**

Cg1 is the centroid of the C25–C30 ring.

**Table:**

*D*—H···*A*	*D*—H	H···*A*	*D*···*A*	*D*—H···*A*
O3—H3···O1	0.84	2.49	3.142 (2)	136
O3—H3···N1	0.84	1.87	2.573 (2)	140
O6—H6···O5	0.84	2.20	2.877 (3)	137
O6—H6···N3	0.84	1.93	2.620 (3)	139
C10—H10···Cg1^ii^	0.95	2.97	3.863 (2)	157

Symmetry codes: (ii) −*x*, −*y*+1, −*z*.
